# Effects of Starter Concentration and Fermentation Time on Microbiological, Physicochemical, Nutritional, and Sensory Properties of Young Coconut Drink–Based Yogurt

**DOI:** 10.1155/ijfo/8077012

**Published:** 2026-09-28

**Authors:** Jeallyza Muthia Azra, Novelina Novelina, Zuraidah Nasution

**Affiliations:** ^1^ Department of Nutrition, Faculty of Public Health, Universitas Andalas, Padang, West Sumatera, Indonesia, unand.ac.id; ^2^ Department of Food and Agricultural Product Technology, Faculty of Agricultural Technology, Universitas Andalas, Padang, West Sumatera, Indonesia, unand.ac.id; ^3^ Faculty of Medicine and Nutrition, IPB University, Bogor, West Java, Indonesia, ipb.ac.id

**Keywords:** lactic acid fermentation, mineral bioaccessibility, physicochemical properties, plant-based yogurt, sensory acceptability, young coconut drink

## Abstract

The development of plant‐based yogurt alternatives continues to expand in response to lactose intolerance, sustainability concerns, and demand for nondairy fermented products. Young coconut drink, derived from a mixture of coconut water and young coconut flesh, has been reported to contain fermentable sugars, minerals, and bioactive compounds that support microbial growth, suggesting its potential as a fermentation substrate. However, its fermentation characteristics remain underexplored. This study evaluated the effects of starter culture concentration (0.8% and 1%) and fermentation time (8 and 10 h) on the microbiological, physicochemical, nutritional, mineral, and sensory properties of young coconut drink–based yogurt. Yogurt was produced using *Lactobacillus delbrueckii* subsp. *bulgaricus* and *Streptococcus thermophilus* at 45°C. All formulations achieved lactic acid bacteria viability above 10^7^ CFU/g, indicating adequate LAB viability after fermentation. Starter concentration significantly influenced acidification, viscosity, proximate composition, carbohydrate utilization, and sensory acceptability, whereas fermentation time exerted a secondary effect. The formulation containing 1% starter fermented for 10 h exhibited the most balanced profile, combining desirable texture, acidity, and overall acceptability. This selected formulation showed a fatty acid profile dominated by medium‐chain fatty acids, particularly lauric acid, a diverse amino acid profile characteristics of the plant‐based matrix, and high mineral bioaccessibility (> 90%) for potassium, magnesium, and iron as determined by an in vitro gastrointestinal digestion model. Overall, young coconut drink–based yogurt demonstrates potential as a plant‐based fermented beverage derived from locally available resources. However, further studies are required to enhance its nutritional profile and sensory characteristics.

## 1. Introduction

Plant‐based fermented foods continue to gain global attention as lactose intolerance, cow′s milk protein allergy (CMPA), and sustainability considerations increasingly influence consumer dietary choices. Fermentation is widely recognized as an effective bioprocess to improve the sensory characteristics, nutritional quality, and stability of plant‐based substrates, making it a promising approach for developing yogurt alternatives [1, 2]. Among these products, plant‐based yogurt has emerged as one of the fastest‐growing categories, stimulating the exploration of novel plant substrates suitable for fermentation [3, 4].

Coconut *(Cocos nucifera L.)* is an abundant tropical resource widely used for food and beverage production and increasingly explored for plant‐based fermentation. Coconut water, in particular, contains natural fermentable sugars (glucose and fructose), electrolytes, amino acids, and bioactive compounds, making it a promising substrate for lactic fermentation [5–8]. However, its very low protein and fat levels limit gel formation and viscosity, resulting in weak texture in yogurt‐type products. To address these limitations, previous studies have demonstrated that combining coconut water with young coconut flesh can increase total solids, fiber content, and viscosity, thereby improving the structural stability and sensory characteristics of coconut‐based beverages [9–11]. In addition, powdered young coconut drinks derived from a water and coconut flesh demonstrated hypoglycemic and body‐weight stabilizing effects in diabetic rat models, indicating potential functional health benefits of the substrate [12]. Building on these findings, a young coconut drink comprising young coconut water and coconut flesh, offers enhanced fermentability for yogurt development due to its improved solids and fiber composition.

Fermentation performance in plant‐based substrates is strongly influenced by starter culture concentration, fermentation time, and other factors such as substrate composition, LAB strains, sugar availability, and storage conditions [13–17]. Lactic acid bacteria (LAB), particularly *Streptococcus thermophilus* and *Lactobacillus delbrueckii* subsp. *bulgaricus*, govern acidification rate, flavor development, protein breakdown, and production of exopolysaccharides (EPS), all of which determine the final texture, acidity, and sensory perception of yogurt [18, 19]. Higher inoculum levels may accelerate acid production but can also induce metabolic stress and reduce bacterial viability, whereas insufficient fermentation time may result in poor texture and unstable acidity ([13, 18, 20, 21]). Optimizing these two parameters is therefore critical for achieving desirable microbiological, physicochemical, and sensory attributes in plant‐based yogurt products.

Although soy, oat, almond, rice, or coconut milk have been extensively used as a base for nondairy yogurt [18, 22, 23], studies using young coconut drink, particularly the combined water–flesh matrix, are still very limited. Moreover, no previous research has systematically examined how variations in starter concentration and fermentation duration interact to influence microbiological viability, proximate composition, mineral profile, and sensory acceptability in such a substrate. This presents an important research opportunity not only to expand the applications of coconut‐based fermentation but also to improve value addition for local coconut commodities.

From a sustainability perspective, developing yogurt from young coconut resources aligns with the Sustainable Development Goals (SDGs), particularly SDG 9 (Industry, Innovation and Infrastructure), by supporting local food processing innovation, and SDG 15 (Life on Land), through promoting the sustainable use of biodiversity‐rich crops such as coconut. Utilizing locally abundant materials also reduces dependency on imported dairy products and strengthens community‐based food value chains.

Therefore, this study aimed to evaluate the effects of starter culture concentration and fermentation time on the microbiological viability, physicochemical properties, mineral composition, and sensory acceptance of yogurt produced from a young coconut drink material. The findings are expected to provide new insights into optimizing fermentation conditions for coconut‐based yogurt and contribute to the development of sustainable, locally sourced, plant‐based functional food products.

## 2. Material and Methodology

### 2.1. Raw Materials

Hybrid young coconuts (*C. nucifera* L.) at approximately 6 months of maturity were obtained from a local supplier in Depok, West Java, Indonesia. Coconut water and soft coconut flesh were manually separated and used as the primary raw materials. This maturity stage was selected based on previous studies reporting favorable physicochemical and sensory characteristics of young coconut drinks produced from hybrid varieties [10, 11].

A commercial freeze‐dried yogurt starter culture containing *S. thermophilus* and *L. delbrueckii* subsp. *bulgaricus* (commercial sachet, Yogourmet, Canada) was used for fermentation. Coconut sugar (Daribumi, Indonesia) was incorporated as a natural sweetener and fermentable carbohydrate source. All chemicals and reagents used for analysis were of analytical grade.

### 2.2. Preparation of Young Coconut Drink Yogurt (YCDY)

Young coconut water and coconut flesh were blended at a ratio of 2:1 to obtain a homogeneous young coconut drink base. The mixture was pasteurized at 80°C–85°C for 5–10 min to ensure microbial safety and improve matrix stability [24]. Coconut sugar (2%) was added after the heat treatment, then rapidly cooled at 45°C to fermentation temperature.

The cooled substrate was inoculated with commercial yogurt starter culture at concentrations of 0.8% and 1.0% *w/w*, following the manufacturer′s recommendation. The viable cell concentration of the commercial starter was not specified on the product label. Fermentation was carried out at 45°C for 8 and 10 h in sterilized glass bottles covered with lids to minimize contamination under semianaerobic conditions. After fermentation, yogurt samples were transferred into plastic containers with plastic lids and stored under refrigerated conditions at 4°C until further analysis.

### 2.3. Experimental Design

The experiment followed a completely randomized factorial design with two factors: starter concentration (0.8% and 1.0% *w*/*w*) and fermentation time (8 and 10 h). All treatments were prepared in triplicate. The interaction effects of fermentation time and starter concentration were evaluated for all measured parameters. The selected starter concentrations and fermentation times were based on preliminary trials. Although only two levels were evaluated for each factor, these conditions were considered sufficient for preliminary assessment of fermentation performance and product characteristics.

### 2.4. Microbial Analysis

The enumeration of LAB was conducted using de Man, Rogosa, and Sharpe (MRS) agar (Merck, Germany) as a general medium for total LAB enumeration. Although M17 agar is more selective for *S. thermophilus*, MRS agar was selected to assess overall LAB viability in the fermented product [25]. Ten grams of each yogurt sample were aseptically homogenized with 90 mL of sterile 0.85% (*w*/*v*) physiological saline solution. Serial decimal dilutions were prepared up to 10^−8^, and 1 mL aliquots from appropriate dilutions were pour‐plated onto MRS agar.

The inoculated plates were incubated at 30°C for 72 ± 3 h under aerobic conditions following ISO 15214:1998 for the enumeration of mesophilic LAB. After incubation, colonies were enumerated and expressed as colony‐forming units per gram (CFU/g). Representative colonies were subjected to Gram staining and catalase testing, and only gram‐positive and catalase‐negative colonies were considered LAB. The present study focused on total LAB viability rather than selective quantification of individual starter microorganisms.

### 2.5. Proximate Composition

The proximate composition of samples was determined according to standard procedures of the Association of Official Analytical Chemists [26] with minor adjustments for liquid fermented products. Moisture content was determined using the oven‐drying method (AOAC Official Method 930.15). Approximately 3–5 g of each sample was placed in a hot air oven and dried at 105°C until a constant weight was achieved. Ash content was measured gravimetrically following AOAC Official Method 930.05 by incinerating dried samples in a muffle furnace at 550°C until white or light‐gray ash was obtained.

Protein content was determined using the Kjeldahl method (AOAC Official Method 978.04). Samples were digested using a semiautomatic Kjeldahl digestion system (DT 208 Digestor, FOSS Analytical, Denmark), followed by distillation using a Kjeltec system (KT 200, FOSS Analytical, Denmark). The distilled ammonia was titrated with standard acid to determine total nitrogen content, and crude protein was calculated using a nitrogen‐to‐protein conversion factor of 6.25. Fat content was analyzed using the Soxhlet extraction method (AOAC Official Method 930.09) with a semiautomatic Soxhlet extraction system (ST 243 Soxtec, FOSS Analytical, Denmark).

Crude fiber content was determined by the acid–alkali hydrolysis method using a semiautomatic crude fiber extraction system (Fibretec System, Tecator, Sweden), following AOAC guidelines. The residue obtained after digestion was dried, weighed, and used to calculate crude fiber content. Carbohydrate content was calculated by difference, using the following equation:

(1) Carbohydrate %=100−protein %+fat %+ash %+moisture %⋯

### 2.6. Physicochemical Analyses

pH values were measured using a calibrated digital pH meter (Ohaus Starter 3100, United States) at room temperature. Titratable acidity was determined according to AOAC procedures and expressed as milliequivalents per 100 g of sample. Total solids content was determined gravimetrically by drying samples at 105°C to constant weight. Viscosity was measured using a rotational viscometer (Daihan Scientific WVS‐2 M Digital Rotary Viscometer, Korea) under controlled temperature conditions. Measurements were performed after sample equilibration to minimize shear effects and expressed in centipoise (cP). Color parameters (L^*^, a^*^, b^*^) were measured using a portable chroma meter (CR‐400; Konica Minolta Sensing, Inc., Osaka, Japan) operating under the CIE L*a*b^*^ color system. The instrument was calibrated using a standard white reference plate prior to analysis.

### 2.7. Mineral Analysis

Mineral contents, including iron (Fe), calcium (Ca), sodium (Na), potassium (K), and magnesium (Mg), were quantified using a wet digestion (wet ashing) procedure following AOAC [26] guidelines. The concentrations of minerals were subsequently determined using an atomic absorption spectrophotometer (Agilent Technologies, 200 Series AA). Sample pH was measured at ambient temperature using a calibrated digital pH meter.

### 2.8. Sensory Evaluation

Sensory evaluation was conducted by 35 untrained adult panelists, recruited from students and staff of the Department of Nutrition, Faculty of Public Health, Universitas Andalas, Indonesia. This study involving human participants was reviewed and approved by the Research Ethics Committee Faculty of Medicine, Universitas Andalas, Indonesia (Ethical Approval No.: 11/UN.16.2/KEP‐FK/2026). All procedures were carried out in accordance with the ethical principles outlined in the Declaration of Helsinki for research involving human subjects. Written informed consent was obtained from all participants prior to their participation in the sensory evaluation. All panelists were adults, and no minors were involved in this study.

Sensory evaluation was performed under controlled conditions. Panelists were instructed to rinse their mouths with water between samples to minimize carryover effects. Product acceptability was assessed using a 9‐point hedonic scale (1 = *dislike extremely*; 9 = *like extremely*), evaluating color, aroma, texture, taste, aftertaste, and overall acceptability. A commercial yogurt drink was included as a reference product. Samples were coded with random three‐digit numbers and presented to panelists in a randomized order.

### 2.9. Amino Acid Profile

The amino acid profile of the selected formulation was determined using high‐performance liquid chromatography (HPLC). Samples were hydrolyzed with 6 mol/L HCl under nitrogen atmosphere at 110°C for 24 h, followed by derivatization prior to chromatographic separation. Individual amino acids were identified and quantified by comparison with external standards [11].

### 2.10. Fatty Acid Composition

Fatty acid composition was analyzed by gas chromatography (GC‐2010 Plus) following lipid extraction, hydrolysis, and methylation to obtain fatty acid methyl esters (FAME). Separation was performed using a capillary column, and fatty acids were identified based on retention times compared with FAME standards. Results were expressed as percentage of total fatty acids [11].

### 2.11. Mineral Bioaccessibility

Mineral bioaccessibility of K, Mg, and Fe was determined using the standardized static in vitro gastrointestinal digestion method according to the INFOGEST 2.0 protocol described by Minekus et al., [27], with minor modifications for fermented liquid samples. The digestion procedure consisted of sequential oral, gastric, and intestinal phases using simulated salivary fluid (SSF), simulated gastric fluid (SGF), and simulated intestinal fluid (SIF), respectively.

During the gastric phase, samples were adjusted to pH 3.0 and incubated with pepsin at 37°C for 2 h. Subsequently, intestinal digestion was performed at pH 7.0 using pancreatin and bile salts at 37°C for 2 h. Following digestion, samples were centrifuged and the soluble fraction was collected for mineral analysis. Mineral bioaccessibility was expressed as the percentage of soluble mineral relative to total mineral content.

### 2.12. Statistical Analysis

All data were expressed as mean ± standard deviation. Statistical analysis was performed using two‐way analysis of variance (ANOVA) followed by Duncan test to evaluate using SPSS 16.0 program. Differences among means were considered significant at *p* < 0.05.

## 3. Results and Discussion

### 3.1. Microbial Viability of LAB

The viable counts of LAB in YCDY ranged from 7.85 × 10^7^ to 1.40 × 10^8^ CFU/g (Table 1), exceeding the Codex minimum requirement for fermented milk products (≥ 10^6^ CFU/g) [28]. Two‐way ANOVA revealed a significant effect of starter concentration (*p* < 0.05), whereas fermentation time showed no significant effect and no interaction was detected.

**Table 1:** Viable lactic acid bacteria (LAB) counts of young coconut drink yoghurt.

Treatment	LAB (CFU/g)
S0.8 F8	1.40 × 10^8^ ± 1.41 × 10^7^ ^aA^
S1 F8	7.85 × 10^7^ ± 1.91 × 10^7^ ^bA^
S0.8 F10	1.15 × 10^8^ ± 7.07 × 10^6^ ^aA^
S1 F10	9.40 × 10^7^ ± 7.07 × 10^6^ ^bA^

*Note:* Different lowercase letters indicate significant differences between starter concentrations within the same fermentation time (*p* < 0.05). Uppercase letters indicate differences between fermentation times within the same starter level (*p* < 0.05). S0.8 F8: yoghurt with 0.8% *w*/*w* starter, fermented 8 h. S1 F8: yoghurt with 1% *w*/*w* starter, fermented 8 h. S0.8 F10: yoghurt with 0.8% *w*/*w* starter, fermented 10 h. S1 F10: yoghurt with 1% *w*/*w* starter, fermented 10 h.

Formulations inoculated with 0.8% starter consistently exhibited higher LAB viability than those with 1%. This inverse pattern aligns with established fermentation kinetics in plant‐based substrates, where higher inoculum levels accelerate acidification and cause a rapid decline in pH, leading to early onset of acid stress that inhibits further LAB multiplication [17, 21]. Increased inoculation also intensifies nutrient competition, especially in matrices with limited nitrogen and low buffering capacity, thereby shortening the exponential growth phase [18, 20]. This behavior is consistent with observations in rice, oat, and wheat substrates, where higher inoculum levels resulted in earlier pH drop and reduced final cell counts [16].

The observed LAB counts were lower than typical dairy yogurts, a trend commonly reported for plant‐based systems due to substantially lower protein and carbohydrate availability compared with milk [16, 29]. Although young coconut drink contains fermentable sugars (glucose and fructose), its low nitrogen content restricts biomass formation despite adequate carbohydrate supply [5]. All formulations in this study also incorporated 2% coconut sugar, which provides additional sucrose and reducing sugars that support early acidification; however, carbohydrate enrichment alone does not overcome the nitrogen limitation of the matrix and therefore does not substantially increase LAB proliferation. The addition of young coconut flesh contributes small amounts of micronutrients and fiber but does not meaningfully improve buffering capacity or protein content, resulting in limited support for extended microbial growth [16]. Similarly, Moiseenko et al. [16] reported that plant‐based substrates with low nitrogen availability showed strong carbohydrate‐driven fermentation but weak biomass accumulation due to limited amino nitrogen.

The absence of significant differences between 8 and 10 h of fermentation suggests that the substrate reaches a metabolic plateau shortly after acidification reaches its endpoint. Similar stagnant growth profiles have been documented in rice‐, oat‐, and almond‐based yogurts, where LAB viability stabilizes once the pH approaches 4.4–4.2 [23, 30]. Moiseenko et al. [16] further demonstrated that once acidification reaches the threshold, LAB viability increases only marginally despite prolonged fermentation, supporting the plateau observed in this study.

Overall, 0.8% *w*/*w* starter provided a more favorable balance between acid development and bacterial growth, producing the highest viable counts. LAB levels remained above 10^7^ CFU/g in all treatments, confirming that young coconut drink is a suitable substrate for producing plant‐based fermented beverages. This microbial activity underpins the physicochemical and sensory characteristics discussed in subsequent sections.

A limitation of this study is that baseline measurements, including initial LAB counts, pH, and titratable acidity (*t* = 0 h), were not determined. Therefore, the present findings primarily reflect endpoint fermentation characteristics rather than microbial growth kinetics throughout the fermentation process.

### 3.2. Proximate Composition

The proximate composition of YCDY is presented in Table 2. Moisture content ranged from 88.08%–88.41%, whereas ash content ranged from 0.62%–0.66%, with no significant differences among treatments (*p* > 0.05). These results are higher than those typically reported for rice‐ or almond‐based yoghurts, which contain 82.97%–84.57% moisture and 0.21%–0.22% ash ([23, 31]), reflecting the naturally high water and ash content of young coconut drink [10]. Conversely, the values obtained in this study were slightly lower than those reported in another young coconut–based yogurt formulation, which contained 90.20%–91.45% moisture and 0.33%–0.75% ash [15].

**Table 2:** Proximate composition of young coconut drink yoghurt.

Treatment	Ash (%)	Moisture (%)	Fat (%)	Protein (%)	Crude fiber (%)	Carbohydrate (%)	Energy (kcal/100 g)
S0.8 F8	0.64 ± 0.01^a^	88.36 ± 0.20^a^	3.42 ± 0.02^a^	0.84 ± 0.01^ab^	6.29 ± 0.03^b^	0.45 ± 0.19^b^	35.98 ± 1.04^ab^
S1 F8	0.64 ± 0.01^a^	88.21 ± 0.20^a^	2.42 ± 0.02^d^	0.87 ± 0.01^a^	5.56 ± 0.04^c^	2.29 ± 0.23^a^	34.48 ± 1.07^b^
S0.8 F10	0.62 ± 0.00^a^	88.41 ± 0.20^a^	2.62 ± 0.01^c^	0.81 ± 0.01^b^	6.76 ± 0.04^a^	0.77 ± 0.16^b^	29.88 ± 0.66^c^
S1 F10	0.66 ± 0.01^a^	88.08 ± 0.17^a^	2.75 ± 0.03^b^	0.74 ± 0.01^c^	5.32 ± 0.05^d^	2.46 ± 0.27^a^	37.57 ± 0.79^a^

*Note:* Values within columns with different superscript letters are significantly different (*p* < 0.05). S0.8 F8: yoghurt with 0.8% *w/w* starter, fermented 8 h. S1 F8: yoghurt with 1% *w/w* starter, fermented 8 h. S0.8 F10: yoghurt with 0.8% *w/w* starter, fermented 10 h. S1 F10: yoghurt with 1% *w/w* starter, fermented 10 h.

The stability of moisture and ash across treatments indicates that fermentation conditions (starter concentration and time) exert minimal influence on water distribution and total mineral content in this matrix. This pattern has also been observed in other plant‐based yoghurts made from oat, almond, or cereal substrates, where fermentation does not significantly alter moisture or ash due to the weak protein–fat network and the absence of water‐binding casein micelles [16, 23]. Thus, the behavior of YCDY aligns with the general characteristics of plant‐based fermentation systems.

Fat content varied significantly among treatments (*p* < 0.05), ranging from 2.42% to 3.42%, with the highest value observed in the S0.8 F8 formulation. These levels were lower than those reported for almond‐based yogurt (3.47%–4.60%; [31]) but higher than rice‐based yogurt (0.37%–0.65%; [15, 23]). The fat fraction in YCDY originates primarily from the young coconut flesh incorporated into the formulation, as coconut water itself contains negligible lipid content. Fat globules are known to contribute to the structural and textural properties of plant‐based yogurts by participating in the gel network and enhancing creaminess [20]. This aligns with consumer expectations, as previous studies show that consumers tend to prefer yogurts made with low‐fat milk rather than nonfat formulations due to their better mouthfeel, creaminess, and overall acceptability [32].

Protein levels were low across all formulations (0.74%–0.87%), consistent with the naturally low protein content of young coconut drink (*p* < 0.5*%*) and limited contribution of young coconut flesh. Formulations inoculated with 1% starter showed significantly lower measurable protein, particularly at 10 h of fermentation. This reduction may be attributed to enhanced proteolytic activity of *L. delbrueckii* subsp. *bulgaricus* and *S. thermophilus*, which rely on extracellular proteases to compensate for nitrogen limitations in plant‐based matrices [18]. Moreover, rapid acidification in high‐inoculum conditions can increase protein precipitation and incorporation into microgel structures, thereby reducing protein solubility during Kjeldahl determination [33]. Similar trends of reduced measurable protein under high inoculum densities have been reported in other plant‐based fermented products [34, 35].

Crude fiber content ranged from 5.32%–6.76% and differed significantly among treatments, with higher values observed at the 0.8% starter level, particularly in the S0.8 F10 formulation. The crude fiber present in YCDY originates from young coconut flesh, which is rich in insoluble dietary fibers such as cellulose and hemicellulose [36, 37]. Lower fiber values in the 1% starter formulations may reflect greater acid‐induced softening or partial solubilization of polysaccharide fractions under more intense fermentation conditions, a phenomenon previously reported in plant‐based fermentations [38, 39]. The modest increase in fiber content at longer fermentation time for the 0.8% starter may also be associated with a concentration effect resulting from carbohydrate depletion while moisture remained stable.

Calculated carbohydrate content ranged from 0.45% to 2.46% and differed significantly among treatments (*p* < 0.05), with consistently higher values observed in formulations inoculated with 1% starter. Because carbohydrate was determined by difference, these values reflect not only residual sugars but also soluble fermentation metabolites, including organic acids, oligosaccharides, and microbial EPS. Higher starter concentrations have been associated with intensified metabolic activity and greater accumulation of such metabolites in plant‐based fermentation systems, which can elevate calculated carbohydrate values without indicating reduced sugar utilization [20]. In addition, the young coconut matrix contains readily fermentable sugars that support rapid early metabolism, contributing to distinct conversion patterns between starter concentrations and fermentation times [5].

Energy values ranged from 29.88 to 37.57 kcal/100 g and reflected the combined contributions of fat and calculated carbohydrate fractions. The S1 F10 formulation exhibited the highest energy value, consistent with its higher residual carbohydrate and moderate fat content, whereas S0.8 F10 showed the lowest energy due to more extensive substrate utilization. Overall, these results indicate that starter concentration exerted a stronger influence than fermentation duration on the nutritional composition of YCDY, with lower inoculum levels supporting more balanced substrate utilization. These compositional trends are consistent with the microbiological patterns observed and help explain the physicochemical and sensory characteristics discussed in subsequent sections.

### 3.3. Mineral Composition

The mineral composition of YCDY is presented in Table 3. Overall, the fermentation treatments significantly influenced the availability of certain minerals, particularly Fe, Ca, and Na, whereas K and Mg remained relatively stable across formulations.

**Table 3:** Mineral composition of young coconut drink yoghurt.

Treatment	Fe (mg/100 g)	Ca (mg/100 g)	K (mg/100 g)	Mg (mg/100 g)	Na (mg/100 g)
S0.8 F8	0.24 ± 0.00^d^	20.64 ± 0.34^b^	196.93 ± 6.31^a^	17.90 ± 0.40^a^	7.86 ± 0.25^a^
S1 F8	0.34 ± 0.00^b^	20.43 ± 0.61^b^	194.26 ± 5.62^a^	17.69 ± 0.23^a^	5.90 ± 0.11^b^
S0.8 F10	0.31 ± 0.01^c^	17.85 ± 0.54^c^	201.02 ± 0.69^a^	17.74 ± 0.40^a^	6.13 ± 0.03^b^
S1 F10	0.35 ± 0.00^a^	22.51 ± 0.14^a^	191.29 ± 1.37^a^	17.66 ± 0.17^a^	4.74 ± 0.01^c^

*Note:* Values within columns with different superscript letters are significantly different (*p* < 0.05). S0.8 F8: yoghurt with 0.8% *w/w* starter, fermented 8 h. S1 F8: yoghurt with 1% *w/w* starter, fermented 8 h. S0.8 F10: yoghurt with 0.8% *w/w* starter, fermented 10 h. S1 F10: yoghurt with 1% *w/w* starter, fermented 10 h.

Fe content increased significantly with fermentation time, reaching the highest value in the S1 F10 formulation (0.35 mg/100 g). This increase may be attributed to fermentation‐induced release of minerals bound to plant cell wall components or organic complexes. Lactic acid fermentation is known to enhance mineral solubility by reducing pH and degrading chelating compounds such as phytates, thereby increasing extractable Fe [18, 40, 41]. In contrast to dairy yoghurt, which is typically a poor source of Fe, plant‐based fermented products often exhibit higher Fe availability due to the intrinsic mineral profile of the raw materials and fermentation‐mediated release mechanisms [40, 42, 43].

Ca content also showed significant variation, with the highest concentration observed in S1 F10 (22.51 mg/100 g). Although these values remain substantially lower than those of dairy yoghurt (≈159 mg/100 g), the observed increase with longer fermentation time suggests improved mineral extractability rather than net mineral gain [40, 42]. Acidification during fermentation can dissociate Ca from insoluble complexes and promote its migration into the soluble phase, as reported in other fermented foods [44–46, 47]. The presence of coconut flesh may additionally contribute minor amounts of Ca, although its role is likely secondary compared with pH‐driven solubilization.

K was the most abundant mineral in all formulations (191–201 mg/100 g) and did not differ significantly among treatments (*p* > 0.05). This observation indicates that, under the fermentation conditions applied in this study, K content remained relatively stable. Young coconut drink is naturally rich in K, which is predominantly present in soluble ionic form (K^+^), contributing to its role as an electrolyte‐rich beverage [11, 48]. However, K stability during fermentation is known to be strain‐ and process‐dependent. Kantachote et al. [49] reported a significant reduction in K during fermentation of mature coconut water by *Lactobacillus plantarum* DW12, attributed to microbial uptake during growth. The absence of significant K depletion in the present study may therefore be related to differences in starter culture composition, shorter fermentation time, and substrate characteristics.

Mg content also remained stable across treatments (17.66–17.90 mg/100 g), suggesting that fermentation did not substantially alter its extractability within the matrix. Similar stability of Mg during lactic fermentation has been reported in coconut‐based and plant‐derived fermented beverages [49].

Na content decreased significantly with increasing starter concentration and fermentation time, from 7.86 mg/100 g in S0.8 F8 to 4.74 mg/100 g in S1 F10. This reduction may be associated with microbial utilization or redistribution of Na ions during fermentation and acidification. Lower Na levels are considered nutritionally favorable, as reduced Na intake is associated with improved cardiovascular health outcomes [50]. The relatively low Na content of YCDY further supports its positioning as a healthier fermented beverage alternative compared with many commercial dairy yoghurts [42].

Collectively, the mineral profile of YCDY highlights the nutritional potential of young coconut–based fermented products, particularly due to their K content and the presence of Fe whose extractability may be enhanced through fermentation. The observed variations in Ca and Fe across fermentation treatments underscore the role of lactic acid fermentation in improving mineral extractability rather than altering total mineral content. In contrast, the relative stability of K and Mg reflects the intrinsic mineral composition of young coconut drink as the primary substrate. These mineral characteristics are consistent with previous reports on young coconut–based beverages, which describe a matrix rich in essential minerals and relatively low in Na [11], and they complement the microbiological and proximate findings, contributing to the overall functional value of the product.

### 3.4. Physicochemical Properties

The physicochemical properties of YCDY are presented in Tables 4 and 5. Overall, fermentation conditions significantly influenced pH, total solids, viscosity, and color attributes, reflecting the metabolic activity of LAB and the unique composition of the young coconut matrix.

**Table 4:** pH and titratable acidity of young coconut drink yoghurt.

Treatment	pH	Acidity (meq/100 g)
S0.8 F8	4.06 ± 0.00^a^	9.92 ± 0.22^a^
S1 F8	4.01 ± 0.01^b^	9.82 ± 0.25^a^
S0.8 F10	4.02 ± 0.01^b^	9.87 ± 0.04^a^
S1 F10	3.98 ± 0.01^c^	10.03 ± 0.23^a^

*Note:* Values within columns with different superscript letters are significantly different (*p* < 0.05). S0.8 F8: yoghurt with 0.8% *w/w* starter, fermented 8 h. S1 F8: yoghurt with 1% *w/w* starter, fermented 8 h. S0.8 F10: yoghurt with 0.8% *w/w* starter, fermented 10 h. S1 F10: yoghurt with 1% *w/w* starter, fermented 10 h.

**Table 5:** Total solids, viscosity, and color of young coconut drink yoghurt.

Treatment	Total solids (%)	Viscosity (cP)	Color		
			L^*^	a^*^	b^*^
S0.8 F8	11.44 ± 0.30^b^	51800.00 ± 5436.91^c^	76.40 ± 5.76^a^	2.25 ± 0.23^b^	6.86 ± 0.50^a^
S1 F8	12.04 ± 0.11^a^	71600.00 ± 12400.00^ab^	73.68 ± 3.16^a^	2.91 ± 0.45^ab^	6.90 ± 0.37^a^
S0.8 F10	12.11 ± 0.05^a^	63466.67 ± 4936.93^bc^	73.79 ± 4.06^a^	2.53 ± 0.48^ab^	6.38 ± 0.22^a^
S1 F10	12.16 ± 0.08^a^	81733.33 ± 10216.33^a^	77.41 ± 2.15^a^	3.19 ± 0.58^a^	8.54 ± 2.22^a^

*Note:* Values within columns with different superscript letters are significantly different (*p* < 0.05). S0.8 F8: yoghurt with 0.8% *w/w* starter, fermented 8 h. S1 F8: yoghurt with 1% *w/w* starter, fermented 8 h. S0.8 F10: yoghurt with 0.8% *w/w* starter, fermented 10 h. S1 F10: yoghurt with 1% *w/w* starter, fermented 10 h.

#### 3.4.1. pH and Titratable Acidity

The pH of YCDC ranged from 3.98 to 4.06, indicating effective acidification across all formulations (Table 4). Samples inoculated with 1% starter exhibited significantly lower pH values than those with 0.8% (*p* < 0.05), regardless of fermentation time, highlighting starter concentration as the primary determinant of acidification kinetics. In contrast, extending fermentation from 8 to 10 h exerted only a minor effect on pH, suggesting that acidification approached equilibrium relatively early in the fermentation process.

Similar patterns have been reported in plant‐based yogurts, where pH is primarily governed by fermentation conditions, bacterial growth dynamics, and substrate availability rather than fermentation time alone [22]. In the present study, the coconut substrate, which is rich in fermentable sugars such as glucose, fructose, and sucrose [51], likely promoted rapid sugar utilization by bacteria, leading to an early pH decline [35], followed by stabilization as nutrient availability decreased and acid stress intensified.

This acidification trend is consistent with the metabolic activity of LAB, which convert readily fermentable sugars such as glucose and fructose into lactic acid, resulting in a rapid decrease in pH [23]. In YCDC, the availability of soluble sugars provides sufficient substrate for early LAB metabolism, promoting rapid acid production during the initial stages of fermentation. Similar substrate‐driven acidification patterns have been reported in other plant‐based yogurts, where higher availability of fermentable carbohydrates supports faster lactic acid formation and a more pronounced early pH decline [22].

Titratable acidity of YCDY ranged narrowly between 9.82 and 10.03 meq/100 g and did not differ significantly among treatments, despite the observed differences in pH (Table 4). This decoupling between pH and titratable acidity has been frequently observed in nondairy yogurts and reflects the low buffering capacity of plant‐based matrices [18, 19]. Unlike dairy yogurt, where casein micelles buffer acid accumulation and allow continued increases in titratable acidity, young coconut drink lacks substantial protein buffering systems, resulting in rapid pH reduction with limited subsequent acid accumulation. Betancourt et al. [22] similarly reported that plant‐based yogurts may reach acidity plateaus despite ongoing fermentation, particularly in substrates with low protein content and limited buffering capacity. These findings support the present observation that higher starter concentration accelerates lactic acid production and pH decline without proportionally increasing titratable acidity.

Importantly, all pH values obtained in this study remained below 4.6, which corresponds to the maximum pH limit defined for yogurt and fermented milk products in regulatory standards [52]. This supports the microbiological stability and safety of the young coconut drink–based yogurt produced in this study.

#### 3.4.2. Total Solids

Total solids content of YCDY ranged from 11.44% to 12.16%, with significantly higher values observed in formulations inoculated with 1% starter (Table 5). This trend indicates that starter concentration exerted a stronger influence on solids distribution than fermentation time. In plant‐based yogurt systems, variations in total solids are primarily associated with changes in soluble fermentation products and matrix organization rather than protein network formation as occurs in dairy yogurt [18].

Fermentation time exerted a comparatively smaller effect on total solids, suggesting that most compositional changes occurred during the early stages of fermentation. Similar patterns have been reported in oat‐, rice‐, and almond‐based yogurts, where inoculum density plays a more critical role than fermentation duration in shaping total solids content [23, 30]. In line with this, Betancourt et al. [22] demonstrated that fermentation conditions strongly govern the transformation of soluble components in plant‐based yogurts through microbial metabolism of available sugars, thereby influencing overall physicochemical properties.

#### 3.4.3. Viscosity

Viscosity values increased with both starter concentration and fermentation time, with the highest viscosity observed in the formulation inoculated with 1% starter and fermented for 10 h. This trend reflects progressive acidification and matrix restructuring during fermentation, which increases resistance to flow as the system transitions from a liquid to a weak gel–like structure [22, 34].

In YCDY, viscosity development is not governed by casein gelation but by interactions among dietary fibers from young coconut flesh, dispersed lipid phases, and fermentation‐induced structural changes. Harper et al. [34] emphasized that plant‐based yogurts with low protein content rely primarily on nonprotein matrix components to develop texture, as plant proteins exhibit limited coagulation capacity compared with dairy systems. Acidification during fermentation promotes closer association among polysaccharides and other matrix components, resulting in increased consistency.

Higher starter concentration likely accelerated acidification kinetics and microbial activity, which facilitated matrix compaction and promoted the production of microbial EPS. EPS‐producing LAB have been widely reported to enhance viscosity and water‐holding capacity in plant‐based yogurts by increasing intermolecular interactions and mimicking the functional role of added hydrocolloids [34, 53, 54]. In this context, EPS may partially compensate for the absence of strong protein networks in YCDY.

In addition, moderate fat levels derived from young coconut flesh may contribute to mouthfeel by acting as a dispersed phase within the fermented matrix, complementing the structural effects of fibers and EPS. Together, these mechanisms explain the observed increase in viscosity with higher starter concentration and extended fermentation time in YCDY.

#### 3.4.4. Color (L^*^, a^*^, b^*^)

Color analysis showed L^*^ values ranging from 69.28 to 82.87, indicating moderate lightness typical of coconut‐based fermented beverages and generally lower than that reported for dairy yogurt (L^*^ ≈74.7) [22, 55]. The relatively lower lightness of YCDY can be attributed to intrinsic differences between plant‐based and dairy matrices. Plant proteins and polysaccharide‐rich systems are known to exhibit darker appearance and reduced light scattering compared with milk proteins, which form highly efficient light‐scattering casein–fat networks [55, 56].

Slight variations in L^*^, a^*^, and b^*^ values were observed across starter concentrations and fermentation times; however, no pronounced shifts in color attributes were detected. This indicates that the applied fermentation conditions did not substantially modify the chromatic properties of the product. Similar color stability has been reported in other plant‐based yogurts, where fermentation primarily affects acidity and texture rather than visual appearance [57]. In nondairy systems, color characteristics are largely governed by the intrinsic composition of the substrate rather than microbial metabolism during fermentation.

Positive a^*^ and b^*^ values reflected mild reddish–yellow hues, which are likely associated with the natural color of young coconut flesh and the addition of coconut sugar prior to fermentation. Unlike dairy yogurt, where yellowness is influenced by riboflavin and carotenoids present in milk fat, coconut‐based systems contain limited those pigments, resulting in less intense chromatic values [55, 58]. The absence of marked browning or discoloration suggests that fermentation of young coconut drink can preserve the natural visual characteristics of the raw material under the conditions applied in this study.

From a sensory perspective, maintaining a stable and uniform color profile is advantageous, as visual appearance strongly influences consumer perception and acceptance of yogurt‐like products. The color stability observed in YCDY supports its suitability as a plant‐based fermented beverage, allowing functional and textural attributes to develop without compromising visual quality.

### 3.5. Sensory Acceptability

The sensory acceptability of YCDY is presented in Table 6. Mean scores for experimental samples ranged from 6.06–6.48 for color, 6.03–6.39 for aroma, 5.61–6.51 for texture, 4.88–5.82 for taste, and 5.09–6.15 for aftertaste. All sensory attributes of YCDY were significantly lower than the commercial yogurt reference (*p* < 0.05), yet remained within the “like slightly” to “like moderately” range on the hedonic scale, indicating overall consumer acceptability.

**Table 6:** Sensory acceptability of young coconut drink yoghurt.

Treatment	Color	Aroma	Texture	Taste	After taste	Overall
S0.8 F8	6.45 ± 1.25^b^	6.18 ± 1.37^b^	5.67 ± 1.52^c^	4.88 ± 1.67^c^	5.09 ± 1.39^c^	5.42 ± 1.39^c^
S1 F8	6.06 ± 1.41^b^	6.18 ± 1.57^b^	5.61 ± 1.49^c^	5.33 ± 1.51^bc^	5.63 ± 1.51^bc^	5.84 ± 1.25^bc^
S0.8 F10	6.39 ± 1.24^b^	6.03 ± 1.57^b^	5.75 ± 1.43^c^	5.30 ± 1.53^bc^	5.64 ± 1.56^bc^	5.67 ± 1.34^c^
S1 F10	6.48 ± 1.22^b^	6.39 ± 1.39^b^	6.51 ± 1.46^b^	5.82 ± 1.64^b^	6.15 ± 1.60^b^	6.39 ± 1.34^b^
Commercial	7.93 ± 0.84^a^	7.69 ± 1.18^a^	7.69 ± 1.13^a^	6.63 ± 1.63^a^	7.06 ± 1.61^a^	7.18 ± 1.44^a^

*Note:* Values within columns with different superscript letters are significantly different (*p* < 0.05). S0.8 F8: yoghurt with 0.8% *w/w* starter, fermented 8 h. S1 F8: yoghurt with 1% *w/w* starter, fermented 8 h. S0.8 F10: yoghurt with 0.8% *w/w* starter, fermented 10 h. S1 F10: yoghurt with 1% *w/w* starter, fermented 10 h.

Among experimental formulations, the S1 F10 sample consistently achieved the highest scores for texture (6.51), taste (5.82), aftertaste (6.15), and overall acceptability (6.39), and differed significantly from samples fermented for shorter durations (*p* < 0.05). In contrast, color and aroma scores did not differ significantly among YCDY formulations (*p* > 0.05), suggesting that fermentation conditions primarily influenced mouthfeel and flavor perception rather than visual or aromatic attributes.

The significant differentiation observed in texture aligns with previous findings in plant‐based yogurts, where consistency is the most sensitive sensory attribute to formulation and processing variables [59]. Similar to reports on peanut‐ and soy‐based yogurts, variations in fermentation conditions may not markedly affect color and flavor but strongly modulate texture and taste [57]. In YCDY, improved texture perception in S1 F10 corresponds with higher viscosity and total solids, supporting the role of acid‐induced structural development, fiber hydration from young coconut flesh, and microbial metabolite formation.

Taste and aftertaste scores were closely associated with acid–sweetness balance. Formulations with extended fermentation exhibited a more pronounced yogurt‐like tang, whereas shorter fermentations retained excessive sweetness and weaker acidity. This pattern is consistent with observations that moderate acidification enhances flavor complexity without causing excessive sourness in nondairy yogurts [57].

Overall, sensory responses corroborated the physicochemical and microbiological findings, indicating that the S1 F10 formulation provided the most balanced combination of texture, flavor, and overall acceptability among the experimental samples; although sensory scores remained lower than those of the commercial dairy yogurt reference. The lower sensory acceptance may be associated with the absence of dairy‐derived flavor compounds, lower sweetness intensity, and the weaker gel structure commonly observed in plant‐based yogurt matrices [34]. Future improvements may include optimization of sweetener composition, incorporation of natural flavoring agents, use of stabilizers or hydrocolloids to enhance texture, and selection of EPS‐producing starter cultures to improve mouthfeel and sensory quality [18, 23, 34].

#### 3.5.1. Amino Acid Profile, Fatty Acid Composition, and Mineral Bioaccessibility of the Selected Formula (S1 F10)

Based on the combined evaluation of nutritional composition and sensory acceptability, the S1 F10 formulation (1% *w*/*w* starter culture, 10 h fermentation) was selected for further analysis. This formulation exhibited favorable energy density, mineral bioaccessibility, and consumer acceptance, indicating its suitability as a functional fermented beverage derived from young coconut.

The total free amino acid content of the selected formulation (S1 F10) was 0.59*%* ± 0.05*%*, reflecting the inherently low protein content of young coconut drink and the limited contribution from young coconut flesh. The amino acid profile was dominated by nonessential amino acids, particularly glutamate (0.11%), aspartic acid (0.06%), and arginine (0.06%), whereas valine (0.06%) was the most abundant essential amino acid detected (Table 7).

**Table 7:** Amino acid profile of S1 F10 YCDY.

Amino acid	Value (%*w*/*w*)
*Essential*	
Threonine	0.03 ± 0.00
Valine	0.06 ± 0.01
Methionine	0.00 ± 0.00
Ileucine	0.03 ± 0.01
Leucine	0.04 ± 0.01
Histidine	0.02 ± 0.00
Lysine	0.03 ± 0.00
Phenylalanine	0.03 ± 0.00
Tryptophane	0.01 ± 0.01
*Nonessential*	
Aspartic acid	0.06 ± 0.01
Serine	0.03 ± 0.00
Glutamate	0.11 ± 0.01
Glycine	0.05 ± 0.01
Alanine	0.04 ± 0.00
Tyrosine	0.02 ± 0.00
Arginine	0.06 ± 0.01
Total	0.59 ± 0.05

The relatively low total free amino acid content is consistent with the plant‐based nature of the substrate and has been reported in coconut‐ and nut‐based fermented beverages [60]. During fermentation, LAB utilize available peptides and free amino acids as nitrogen sources, resulting in partial depletion of the free amino acid pool. Similar reductions in total free amino acids after fermentation have been attributed to microbial metabolism, transamination reactions, enzymatic conversions, and incorporation into secondary metabolites [61–63].

Despite the low total concentration, the presence of diverse free amino acids—particularly glutamate and aspartate—may contribute positively to flavor development, imparting mild umami and savory notes. Previous studies have shown that free amino acids play a key role in sensory perception rather than nutritional protein density in plant‐based yogurts [62, 64]. Overall, the amino acid profile of YCDY reflects substrate limitations and fermentation‐driven nitrogen redistribution rather than protein enrichment.

The fatty acid profile of the selected formulation (S1 F10) was characterized by a predominance of medium‐chain fatty acids (MCFA), which is typical of coconut‐derived lipids. Lauric acid (C12:0) was the major fatty acid (14.18*%* ± 5.97*%*), followed by palmitic acid (C16:0; 14.68*%* ± 1.56*%*), oleic acid (C18:1n9c; 13.57*%* ± 2.64*%*), and myristic acid (C14:0; 4.87*%* ± 2.18*%*) (Table 8). Caprylic (C8:0) and capric acids (C10:0) were also detected, confirming the substantial contribution of young coconut flesh to the lipid fraction.

**Table 8:** Fatty acid composition of S1 F10 YCDY.

Fatty acid	Value (%*w*/*w*)
*Saturated*	
Butyric acid, C4:0	0.27 ± 0.10
Caproic acid, C6:0	0.30 ± 0.03
Caprylic acid, C8:0	1.96 ± 0.50
Capric acid, C10:0	1.37 ± 0.52
Lauric acid, C12:0	14.18 ± 5.97
Myristic acid, C14:0	4.87 ± 2.18
Palmitic acid, C16:0	14.68 ± 1.56
Stearic acid, C18:0	1.76 ± 0.06
*Unsaturated*	
Oleic acid, C18:1n9c	13.57 ± 2.64
Linoleic acid, C18:2n6c	3.00 ± 2.35
Total	55.95 ± 2.59

This fatty acid distribution is consistent with previous studies on coconut‐based beverages and coconut fat, which report high proportions of MCFA, particularly lauric acid, as a defining characteristic of coconut‐derived lipids [7, 11, 65, 66]. Similar profiles have been reported for coconut yogurt alternatives, where MCFA dominate the saturated fatty acid fraction and distinguish these products from dairy yogurts, which are typically richer in long‐chain saturated fatty acids (LCFA) such as palmitic and stearic acids [67]. Moreover, the palmitic acid in this study was lower than other plant‐based yoghurt [68].

Overall, the fatty acid composition of YCDY reflects the intrinsic lipid characteristics of young coconut flesh rather than transformations induced by fermentation, indicating that the nutritional quality of the fat fraction is primarily governed by the raw material used.

Mineral bioaccessibility analysis revealed high digestibility values for K (0.91 ± 0.03), Mg (0.93 ± 0.03), and Fe (0.91 ± 0.07), indicating that more than 90% of these minerals were potentially bioaccessible (Table 9). The high mineral digestibility may be attributed to lactic acid fermentation, which lowers pH and promotes the solubilization of minerals through organic acid production. In addition, young coconut matrices are naturally low in antinutritional factors such as phytates, further enhancing mineral bioaccessibility than other plant‐based yoghurts [43, 69].

**Table 9:** Mineral bioaccesibility of S1 F10 YCDY.

Mineral bioaccessibility	Value (%)
K	0.91 ± 0.03
Mg	0.93 ± 0.03
Fe	0.91 ± 0.07

Overall, the extended nutritional evaluation of the selected S1 F10 formulation demonstrates that YCDY not only achieves desirable microbiological and sensory quality but also provides functional nutritional components, including a diverse amino acid profile, MCFA‐rich lipid composition, and highly digestible essential minerals. These findings reinforce the potential of young coconut drink–based yogurt as a functional fermented beverage that supports food diversification and sustainable utilization of local coconut resources.

## 4. Conclusion

This study demonstrated that young coconut drink supplemented with young coconut flesh can be used as a substrate for producing a plant‐based fermented yogurt‐like product. All formulations achieved total LAB counts above 10^7^ CFU/g after fermentation. Starter concentration showed a greater influence than fermentation time on physicochemical, microbiological, nutritional, and sensory characteristics. The formulation produced with 1% *w*/*w* starter and 10 h fermentation (S1 F10) exhibited the most balanced combination of acidity, viscosity, mineral bioaccessibility, and sensory acceptability. The selected formulation showed a diverse amino acid profile and a fatty acid composition dominated by MCFA, particularly lauric acid. Overall, the findings suggest the potential application of young coconut drink as a locally sourced substrate for plant‐based fermented beverages, although further studies are needed to improve sensory quality and evaluate additional functional properties.

## Author Contributions

Jeallyza Muthia Azra designed and supervised the study, performed data analysis, and wrote the manuscript. Novelina Novelina conducted microbiological analysis and assisted in data interpretation. Zuraidah Nasution performed mineral bioaccessibility analysis and contributed to literature review.

## Funding

This study was financially supported by the Institute for Research and Community Service, Universitas Andalas (456/UN16.19/PT.01.03/PLB/2025).

## Disclosure

All authors approved the final manuscript.

## Conflicts of Interest

The authors declare no conflicts of interest.

## Data Availability

The data that support the findings of this study are available from the corresponding author upon reasonable request.
